# Refining Criteria for Choosing the First-Line Treatment for Real-World Patients with Advanced *ALK*-Rearranged NSCLC

**DOI:** 10.3390/ijms26135969

**Published:** 2025-06-21

**Authors:** Edyta Maria Urbanska, Peter Rindom Koffeldt, Morten Grauslund, Linea Cecilie Melchior, Jens Benn Sørensen, Eric Santoni-Rugiu

**Affiliations:** 1 Department of Oncology, Rigshospitalet, Copenhagen University Hospital, DK-2100 Copenhagen, Denmark; jens.benn.soerensen@regionh.dk; 2 Department of Pathology, Rigshospitalet, Copenhagen University Hospital, DK-2100 Copenhagen, Denmark; peter.rindom.koffeldt@regionh.dk (P.R.K.); morten.grauslund@regionh.dk (M.G.); linea.cecilie.melchior@regionh.dk (L.C.M.); 3 Department of Clinical Medicine, University of Copenhagen, DK-2200 Copenhagen, Denmark

**Keywords:** *ALK* rearrangement, intrinsic resistance, first-line ALK-TKI, de novo co-alterations, IHC/FISH/NGS

## Abstract

Choosing the optimal first-line treatment for patients with advanced non-small cell lung cancer (NSCLC) with anaplastic lymphoma kinase (*ALK*) rearrangements can be challenging in daily practice. Although clinical trials with next-generation ALK-tyrosine kinase inhibitors (TKIs) have played a key role in evaluating their efficacy and safety, which patients benefit from a specific ALK-TKI may still be questioned. The methodological inconsistencies in these trials, which led to the inclusion of different patient populations, appear to have been inadequately addressed. *ALK-*rearranged NSCLC is a heterogeneous disease, and co-existing molecular alterations may affect the outcome. The questions explored in these trials appear insufficient to support a personalized approach to the first-line treatment, while defining long-term responders and early progressors would be clinically useful. This narrative review presents several considerations from oncologists’ and pathologists’ perspectives. We propose defining favorable and unfavorable features, such as histology, type of *ALK* fusion, co-existing molecular alterations, plasma circulating tumor DNA (ctDNA, performance status, and brain metastases, to help identify patients with lower and higher risk of progression. Consequently, the most potent ALK-TKI to date, Lorlatinib, may be considered as the first-line treatment for high-risk patients with unfavorable features, while sequencing of ALK-TKIs may be appropriate for low-risk patients with favorable features. Although ALK signal inhibition is critical in this disease, it may not be sufficient for clinical control due to de novo co-alterations. A more personalized approach to first-line therapy requires consideration of risk factors for each patient.

## 1. Introduction

The recently published 5-year results of the CROWN study are impressive and unprecedented by any comparable data in the field of targeted therapies for non-small cell lung cancer (NSCLC) [1]. This is certainly good news for some patients with advanced *anaplastic lymphoma kinase* (*ALK*)-rearranged NSCLC, as 60% of patients receiving Lorlatinib remained on treatment after five years. This third generation ALK-tyrosine kinase inhibitor (TKI) resulted also in a significant delay in intracranial progression, which is clinically important given the high propensity of *ALK*-rearranged NSCLCs to spread to the central nervous system (CNS) [2]. These results have renewed the debate whether all advanced *ALK*-rearranged NSCLCs should receive Lorlatinib as first-line treatment, sequential treatment with second-generation ALK-TKIs, or even other combinations [3,4,5,6,7,8]. Therefore, we hereby review the literature related to this debate and based on the described histopathological, molecular, and clinical factors, highlight their significance for treatment outcome, defined in the literature as objective response rate (ORR), median progression-free survival (mPFS), and/or overall survival (OS) (Table 1).

Different combinations of these features may help to define the risk of progression for the individual patient and enable a more personalized choice of the first-line treatment. The relevant features are discussed in the next paragraphs.

## 2. How Do Diagnostic Intricacies Affect Interpretation of the Treatment Outcome?

Concordance and discordance between diagnostic methods in phase III studies and in real-world data with ALK-TKIs has been shown to affect treatment outcome, and hereby also revealed different patients’ populations with *ALK* rearrangement. In the ALTA-1L study, Brigatinib-treated patients achieved a mPFS of 24 months, whereas in the ALEX study, patients receiving Alectinib achieved a mPFS of 34 months [6,7]. Comparing the CROWN, ALTA-1L, and ALEX studies should be performed with caution, as their design, diagnostic methods, and endpoints vary and represent different patients’ populations.

### 2.1. ALTA-1L Trial

Inclusion in the ALTA-1L study required *ALK* rearrangement locally assessed by fluorescence in situ hybridization (FISH), which was considered as a core diagnostic approach. However, immunohistochemistry (IHC) or alternative methods were accepted after central confirmation [8]. In total, 58% of patients (*n* = 160) were diagnosed with FISH, 49% with IHC, and 4% (10 patients) by other methods, suggesting that only 11% of patients were diagnosed with more than one method. Yet, *ALK*-FISH-positive cases not confirmed by other methods may result in inclusion of false positive cases without *ALK* rearrangement [9,10]. Despite reported high concordance between ALK-IHC- and *ALK*-FISH-positive cases (75.9%), the discordance between ALK-IHC-positive and *ALK*-FISH-negative cases results in shorter mPFS [11]. On the other hand, in 25% of ALK-IHC-positive cases, FISH could not detect *ALK* rearrangement [12]. The percentage of *ALK*-rearranged tumor cells scored by FISH is also relevant to the outcome, as low percentages correlate with poorer response to ALK-TKIs [13,14,15]. Yet, the ALTA-1L study did not report any correlation between percentage of FISH-positive tumor cells and treatment response. Additionally, in the ALTA-1L, in contrast to the ALEX and CROWN studies, about one-third of patients were pretreated with chemotherapy and received an ALK-TKI as second line, thus representing another patient population, making comparisons among the three trials difficult.

### 2.2. ALEX Trial

In the ALEX study, *ALK*-rearranged tumors were locally diagnosed by IHC followed by central confirmation by ALK-IHC and -FISH [7]. Retrospective analysis revealed longer PFS in patients with IHC-positive/FISH-positive tumors than in those with IHC-positive/FISH-negative tumors (HR 0.37 and 1.33, respectively) [16]. Furthermore, patients with IHC-positive/FISH-positive tumors exhibited higher ORRs when receiving Alectinib (90.6%) than Crizotinib (81.4%), as opposed to patients with IHC-positive/FISH-negative tumors (28.6% vs. 44.4%) [16]. The surprisingly higher response to Crizotinib in the latter group may reflect an inhibitory effect on highly homologous (in their amino acid sequence) targets other than ALK protein, such as ROS1 or MET. Interestingly, post hoc hybrid capture, next-generation sequencing (NGS) was performed in 35/39 patients with IHC-positive/FISH-negative NSCLC, and in 57.1% of them, no *ALK* fusion was identified [16]. Although this could be due to both the post hoc nature of the study and sparse number of *ALK*-rearranged cells caused by tumor heterogeneity, it is in any case in line with previously reported IHC-positive/FISH-negative cases, and the absence of *ALK* transcripts by reverse transcription polymerase chain reaction (RT-PCR) [9,10]. Thus, these methodological discrepancies in assessing *ALK* rearrangement have revealed a group of ALK-IHC-positive patients with worse prognosis and indicated that the discordance between positive ALK-IHC and negative FISH and NGS is itself an unfavorable feature (Table 1). Thus, assuming that the FISH and NGS results were not false negative and that the *ALK* gene was not rearranged, determining ALK status based on IHC alone [1,7,71] might not be sufficient for oncologists in making their therapeutic decision [17]. Indeed, despite being a disputed issue, the possibility that ALK expression may result from molecular alterations other than gene fusion, such as amplification or transcriptional upregulation, cannot be excluded [18,19,20]. As shown in the retrospective analysis of the ALEX trial [7,16], lack of orthogonal verification tests carries the risk that the screened patients’ population may not be the same as the one from multiple tests. Indeed, in the ALEX study, if the positive IHC was not confirmed by FISH and NGS, the response to ALK-TKI was lower [16]. This discordance represents an unfavorable feature (Table 1).

Large cohorts and case series have indicated that 20–30% of FISH-positive cases are IHC- and NGS-negative, which represents another unfavorable discrepancy that is associated with limited response to ALK-TKIs (Table 1) [9,10,11,12,17]. Yet, non-responders can also be observed despite concordance among the three methods [17]. This implies that 100% ORR on an ALK-TKI might be hardly achievable because of intrinsic resistance. One of the highest ORRs (92.8%) was observed with Alectinib-treated patients in the J-ALEX study, in which the concordance with NGS was not evaluated, as *ALK* rearrangement was diagnosed with IHC and FISH or RT-PCR [80].

Importantly, there was no call for NGS analysis of the tumor tissue in the ALEX and ALTA-1L. Nonetheless, both studies were enriched by plasma cell-free DNA (cfDNA) analysis. In the ALEX study, this analysis indicated the prognostic value of median cfDNA concentration and different ORRs of *echinoderm microtubule-associated protein-like 4 (EML4)-ALK* variant (v.) 1 and 3 to Alectinib (90% and 68%, respectively) [72]. In contrast, in ALTA-1L, cfDNA genotyping revealed similar ORR of v.1 and v.3 to Brigatinib, but the mPFS was significantly longer for v.1 than for v.3. [8].

### 2.3. CROWN Trial

In the CROWN study, the median ORRs for *EML4-ALK* v.1 and v.3 were unavailable, but mPFS in patients treated with Lorlatinib was similar and the longest ever reported, i.e., 60.0 and 64.3 months, respectively [1,71]. Thus, the 5-year data for the entire cohort in the CROWN study, despite the non-reached (NR) mPFS in Lorlatinib-treated patients, reflect higher efficacy of this drug as compared to Alectinib and Brigatinib. Indeed, the corresponding mature results of the ALEX trial indicated a mPFS of 34.8 months [7,91]. Notably, the 5-year follow-up data of mPFS in the ALTA-1L has not been reported so far.

The major argument for choosing Lorlatinib as first-line treatment is to use the currently most effective drug among ALK-TKIs [81] and to avoid on-target resistance at progression, especially compound *ALK* mutations, which are more frequent in later lines [40,92]. However, the recent data of the fourth-generation ALK-TKI, NVL-655, demonstrates significant potential for overcoming other ALK-TKIs’ limitations, including intra- and extracranial efficacy in cases with multiple single and compound *ALK* mutations and limited toxicity [93].

Accordingly, in the CROWN study, circulating tumor DNA (ctDNA) analysis was performed in some patients receiving Lorlatinib, revealing bypass aberrations as main resistance mechanisms and better outcome in ctDNA-negative patients. This is similar to plasma genotyping in the ALTA-1L and ALEX studies and represents a favorable feature for lower risk of progression [1,8,41,71,74]. Nevertheless, the three studies differ in patients’ populations, methodologies, and timing for plasma genotyping, thereby becoming difficult to compare [75]. Yet, the main question remains as to which *ALK*-rearranged NSCLC patients may achieve the most benefit from first-line Lorlatinib. In the CROWN study, the first patient was randomized in November 2019. The inclusion criteria required the diagnosis of *ALK* rearrangement by IHC only [1,71], although international guidelines for molecular testing of advanced NSCLC also recommended NGS as a diagnostic method [94,95,96,97]. Certainly, ALK-IHC is considered as a pathognomonic surrogate for *ALK* rearrangement, given that the *ALK* gene is downregulated after the embryonal phase, and only a limited expression of *ALK* mRNA might be found in the small intestine, testis, prostate, and brain [98,99]. However, using positive ALK-IHC alone to diagnose *ALK* rearrangement does not inform whether this represents a fusion/multiple fusions, nor about the specific fusion partner and whether there are genomic co-alterations, all of which may impact the response to ALK-TKIs. Indeed, as mentioned above, ALK-positive immunostaining may also result from rare occurrence of de novo *ALK* amplification [18,19,20]. Finally, the rare cross-reactivity of antibodies against the ALK and ROS1 proteins due to their significant amino acid sequence homology should be considered, as it may potentially affect the specificity of diagnostic tests [100,101,102,103].

### 2.4. NGS

**Figure 1:**
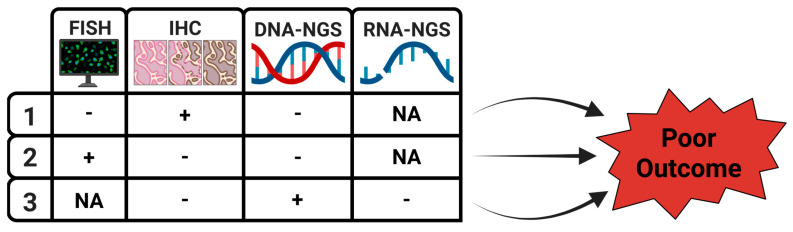
Discordances among current diagnostic methods (FISH, IHC, DNA-NGS, RNA-NGS) that result in poor outcomes. NA: non-applicable, indicates lack of analysis in the citated studies. References for row 1 [9,10,11,12,16,17,18,21,22]. References for row 2 [9,10,11,12,17,19,20,21,22,26]. References for row 3 [17,23,24].

The implementation of NGS analysis on tissue biopsies has allowed us to define various canonical *EML4-ALK* variants and to discover new fusion partners (at least 120) [40,42,43]. Similarly with specific *EML4-ALK* variants, non-canonical *ALK* fusions may determine variable sensitivities to ALK-TKIs [44,45]. Yet, ALK-IHC is critical for confirming whether the *ALK* fusion detected by NGS results in a productive fusion protein, which is the ultimate TKI target [11,17,21,22]. The concordance between ALK-IHC and DNA-NGS is reported to reach 84.5% [11]. This implies that there is a minor proportion of patients with either non-productive fusion or ALK protein coded by mechanisms other than fusions transcripts [18,19,20] who are consequently associated with poor outcomes. Detection of *ALK* fusions and genomic mechanisms of ALK-TKI resistance is also possible on plasma cfDNA [76,77]. However, DNA-based NGS may not always be sufficient to diagnose *ALK* fusions and predict their responsiveness to ALK-TKIs. Indeed, RNA-NGS is more sensitive, as it allows more certain identification of specific, known, and unknown fusion partners [45] and, hence, the detection of 10–14% more *ALK* fusions in tissue samples [23,24,25]. Importantly, finding the fusion transcript confirms the functional level of ALK and demonstrates high concordance with ALK-IHC [26]. Additionally, NGS analysis of plasma cell-free RNA, when feasible, can detect actionable gene fusions with higher sensitivity than cfDNA [78]. Notably, the detection of circulating *ALK* fusion transcripts correlates with a significantly worse outcome [79]. A unique advantage of RNA-NGS is the possibility of excluding those uncommon fusions that are not producing fusion proteins targetable by ALK-TKIs [23,24,25,45,46]. The reasons for unsuccessful fusion gene detection by DNA-NGS may be low coverage of fusion breakpoints, introns missing in the panel design, or repetitive elements in the introns rendering sequence mapping to the genome difficult [25]. For these reasons and given the existence of complex *ALK* rearrangements, the most reliable diagnostic approach for detecting actionable *ALK* fusions would be to combine DNA- and RNA-NGS, along with validation of fusion protein expression as ultimate TKI target by IHC [104]. The lack of concordance between DNA-NGS and RNA-NGS is also linked to poor outcomes (Figure 1).

However, access to various diagnostic methods and, consequently, orthogonal testing validation are not always possible in the real world, as they may be limited by logistic, technical, and economic restrictions [105,106].

Altogether, these diagnostic considerations imply uncertainty regarding the application of the existing data to daily practice. Particularly, cases with diagnostic discrepancy represent an unfavorable feature because of the uncertain or predominantly poorer response to treatment (Figure 1).

## 3. The Importance of Intrinsic Resistance to ALK-TKIs for the First-Line Treatment

Different forms of intrinsic resistance should also be investigated before choosing first-line treatment (Table 2). Indeed, in the CROWN study, after 12 months, 70% of patients receiving Lorlatinib still responded, while the remaining 30% did not, presumably because of intrinsic resistance [1]. *ALK* fusions are much more frequent in adenocarcinomas than other types of NSCLC, yet they are particularly enriched in adenocarcinomas with acinar, solid, or mucinous growth patterns, as well as those containing signet-ring cells. Importantly, the presence of signet-ring cells in adenocarcinomas, in general, is associated with significantly more advanced stages and decreased survival, due to rapid cancer spreading [27,28,29,30,31]. Most patients with *ALK* rearrangement have an adenocarcinoma phenotype, but there are also several real-world data regarding patients with mixed or other phenotypes. The rarely reported *ALK*-rearranged NSCLCs with squamous histology have exhibited inconsistent sensitivity to ALK-TKIs, responding to Ensartinib but not to Alectinib [32,33]. This group represents another therapeutic challenge as it is poorly explored, given that NGS is still not recommended for squamous cell carcinomas [107,108]. Similarly, cases of *ALK*-rearranged large-cell neuroendocrine carcinoma (LCNEC) and atypical carcinoids have been described, with some of them responding to ALK-TKIs; however, the optimal treatment for these patients remains unclear [34,35,36]. Additionally, cases of pulmonary neuroendocrine tumors, including carcinoids, LCNEC, and small-cell lung carcinoma (SCLC) exhibiting ALK-positive immunostaining despite lack of *ALK* fusion or amplification have been reported, implying that the detection of *ALK* fusions in these neoplasms requires confirmation by FISH and/or NGS [35,37]. Interestingly, several cases of SCLCs with *ALK* fusion were reported and showed inconsistent responses to ALK-TKI [38,39]. However, longer follow-up is needed to assess the potential of ALK-TKIs in SCLC.

Beyond histology, different *ALK* fusions and co-existing molecular alterations may a priori determine both sensitivity to ALK-TKIs [45] and shape the clinical course of the disease [46,47], as indicated by recent real-world reports [48,49,50,51]. Rarely reported double or even triple *ALK* fusions generally exbibit sensitivity to ALK-TKIs [52,53]. However, the still-limited and inconsistent data of long-term response do not support multiple fusions as a favorable factor for outcome [54,55,56].

Notably, post hoc plasma genotyping in the CROWN study has revealed that 3.7% of patients treated with Lorlatinib and 5.4% of patients treated with Crizotinib had at baseline ≥ 1 de novo concurrent *ALK* mutations [1]. This is consistent with previous data showing that a minor proportion of ALK-TKI-naïve patients may harbor de novo co-mutations in the *ALK-*kinase domain [60], which may affect the therapeutic outcome [61]. Furthermore, co-existing bypass alterations, such as deletion of the *CDKN2A/B* tumor suppressor gene, may have an impact on the course of the disease by causing an increased tendency for central nervous system (CNS) spread [62]. Moreover, deletion of *CDKN2A/B* is associated with shorter PFS [57]. Retrospective data of patients treated with Crizotinib showed significantly shorter PFS in the group with concurrent gene amplifications [63]. Co-occurrence of *TP53*-mutations at baseline was also reported to reduce the efficacy of ALK-TKIs [57,64,65,74]. This was also observed in the subgroup of plasma-genotyped patients in the CROWN study, with mPFS of 51.6 months in *TP53*-mutated vs. NR in the *TP53* wild-type cases [1]. Furthermore, *TP53* mutations were detected in 57% of early progressors (PFS ≤ 12 months) on Lorlatinib, as compared to 22% in non-progressors [109]. Real-world data shows that particularly detrimental for patients treated with ALK-TKIs, including Lorlatinib, is the co-occurrence of *EML4-ALK* v.3 and *TP53* mutation, resulting in markedly shortened mPFS [58,59,65]. A promising approach combining Alectinib with a proteasome inhibitor to address the significant frequency of *TP53* co-mutations and ALK-TKI resistance has been reported [66]. Finally, other de novo alterations such as *EGFR* and *KRAS* mutations may co-exist and have a negative impact on the response to ALK-TKI [67,68]. However, there is limited data showing that the response may be ALK-TKI specific, and Ensartinib may be efficient in cases of co-alterations like *TP53*, *EGFR*, and *ERBB2* mutations [110]. Similarly, several de novo co-alterations like overexpression of *MET*, *AKT1*, *EGFR*, *MUC21*, and *PTGS2* found in long responders in the eXalt3 trial have not caused resistance to Ensartinib [111]. Another question is whether first-line ALK-TKI together with chemotherapy would also be an option for some patients, as preclinical models suggest that this approach has synergistic effects that may overcome primary TKI resistance [112]. Similarly, the FLAURA2 study displayed that in *EGFR*-mutated NSCLC, combined first-line Osimertinib with chemotherapy led to significantly longer PFS than Osimertinib monotherapy [113]. Although we still have not adequately characterized the patients who derive the greatest benefit from this combination, this is a worthwhile treatment option [114]. Thus, we await with interest the results of the Japanese B-DASH study, which is the only trial of Brigatinib with chemotherapy in patients with untreated, *ALK*-rearranged NSCLC [115]. Importantly, de novo druggable co-alterations, such as *MET* amplification, may also occur and require another approach, such as combining ALK- and MET-TKIs [69]. Moreover, *MET* alterations followed by *NF2* mutations were found to be the most frequent promoters of early ALK-TKI resistance [70].

Clinical factors like performance status (PS) and sex have also been explored in NSCLC patients with *ALK* rearrangement. Clinical benefit from ALK-TKIs was observed in all patients, but the outcome was significantly better in patients with performance status (PS) 0–1 [82,89]. Despite the lack of data regarding sex-specific response, no significant differences were observed between men and women in a systematic review of nine studies with ALK-TKIs [116]. Finally, pharmacological aspects may also be considered for choosing a specific ALK-TKI. The genotypes of cytochromes (CYPs) metabolizing ALK-TKIs and the expression of P-glycoprotein transporting some ALK-TKIs together with the frequent co-medication in NSCLC patients, potentially generating drug interactions, may also affect the outcome [117,118,119,120]. However, alterations in P-glycoprotein and variants of CYPs are not a part of molecular set-up and there is still no data to inform the clinical approach. Table 2 summarizes the above-described factors of intrinsic resistance to ALK-TKIs reported in the literature so far.

**Table 2:** Reported factors of intrinsic resistance to ALK-TKIs. LUAD: lung adenocarcinoma; SqCC: squamous cell carcinoma; LCNEC: large-cell neuroendocrine carcinoma; SCLC: small-cell lung carcinoma. CYPs: cytochromes. * Yet not applicable in the real-world setting.

	Reported Factors of ALK-TKI Intrinsic Resistance	References
**1**	Histology: LUAD, SqCC, LCNEC, SCLC	[27,28,29,30,31,32,33,34,35,36,37,38,39]
**2**	*ALK* fusion partners and *EML4-ALK* variants, double/triple *ALK* fusions	[40,41,42,43,44,45,46,47,48,49,50,51,52,53,54,55,56,57,58,59]
**3**	De novo on-target mutations in *ALK*	[60,61]
**4**	De novo off-target mutations in other oncogenes: *EGFR*, *KRAS*, *MET*	[67,68,70]
**5**	De novo *MET* amplification	[69]
**6**	De novo alterations in tumor suppressor genes: *CDKN2A/B*, *NF2*, *TP53*	[40,41,57,58,59,62,70,110]
**7**	(Genotypes of CYPs and P-glycoprotein transporter) *	[117,118,119,120]

## 4. Different ALK-TKIs May Provide Long-Term Responses, Including Crizotinib

Beyond the impressive results of the CROWN study, there are real-world reports confirming the high efficacy of Lorlatinib, especially with intracranial progression [121,122,123]. However, there is also considerable real-world data showing long-term responses to Brigatinib [124,125,126], Alectinib [83,127,128], and even Crizotinib [84,129]. The recently published retrospective CRIZOLONG study showed that patients with advanced *ALK*-rearranged NSCLC profited from first-line Crizotinib with a median duration of treatment of 43.3 months [84]. In almost all patients, ALK status was determined by either IHC, FISH, or both techniques, though not by NGS. The majority of these long-term responders were characterized by paucisymptomatic and oligometastatic disease without brain metastases. Additionally, two reports of patients treated with first-line Crizotinib and showing complete response for 5 and 6 years were described [85,86]. Nevertheless, the clinical and molecular features of long-term responders to different ALK-TKIs are not fully elucidated. As shown in the CROWN trial, there is even a small population of NSCLC patients with *ALK* rearrangement in whom Crizotinib has sustained consistent response over five years [1], and Lorlatinib might represent an overtreatment. What enables these approximately 10% of patients to reap the benefit of continuing treatment with this first-generation ALK-TKI still remains to be answered, since most data clearly show the better effectiveness of the next-generation ALK-TKIs [1,6,7,8,71,90,91].

## 5. Future Perspectives—Favorable and Unfavorable Factors Should Be Included in Therapeutic Decision

**Figure 2:**
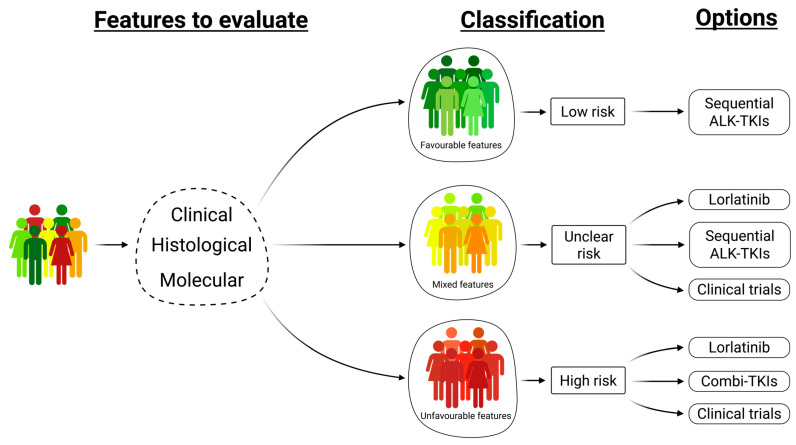
Proposed personalized approach for choosing the first-line treatment for patients with low, uncertain, and high risk of progression based on the clinical, histological, and molecular features illustrated in detail in Table 1.

Biotechnological progress is outpacing our understanding of NSCLC. Five-year-old clinical trials did not consider current challenges because these were not completely understood at the time, and questions, which are relevant today, were not asked. The CROWN, ALTA-1L, and ALEX studies together with real-world data imply that we need to use more molecular information to provide a personalized approach to treatment. It is now increasingly important to use more data, if possible, as well as those generated by multiomics of tissue and plasma samples, to determine the patients’ prognosis and/or response to therapy [130,131]. For clinical application, it is important to define favorable and unfavorable features as they may imply a different risk of progression. As discussed above, the up-front spread to CNS and presence of ctDNA indicate a more aggressive disease, and patients with these features are supposed to significantly profit from Lorlatinib [1,71,87]. The patients with favorable features like canonical *ALK* fusion without *EML4-ALK* v.3, no co-alterations, no CNS metastases, and baseline negative ctDNA suggesting more indolent diseases, may benefit more from sequential treatment without risk of overtreatment. In this context, real-world data showed significantly longer OS in patients receiving multiple lines of ALK-TKI compared with only one ALK-TKI [44,88]. As presented above, discordance between ALK-IHC and *ALK*-FISH is associated with poor outcomes and may thus demonstrate an unfavorable feature [9,10,11,12,16,17,18,19,20,71,87]. As it is not always feasible to perform all these three methods, it seems that the most important is concordance between ALK-IHC and *ALK*-NGS [17,23,26,46,87] (Figure 1). Taking all these considerations into account, we propose to define low-, uncertain-, and high-risk groups based on clinical, histological, and molecular features. The available factors that might be considered relevant for first-line decision-making are proposed in Figure 2.

Additionally, as per best practice standards, other factors such as comorbidities and treatment toxicities should also be considered, as extensively discussed elsewhere [1,4,6,7,8,44,71].

Moreover, a data science-based approach will be needed in the future. We may use the experience from hematology, in which machine learning-based predictive models helped to improve the treatment and provided new knowledge [132]. In this respect, CROWN, ALTA-1L, and ALEX studies provided limited molecular information and no data modeling. In the CROWN trial, the mPFS of patients treated with Crizotinib was somehow shorter (9.3 months) than observed in other phase III trials with ALK-TKIs [1,6,7,8,71,133], and only 5.3 months in the group with *TP53*-mutations [1]. Therefore, we cannot exclude that in the Crizotinib arm of the CROWN study there might have predominantly been patients with poorer prognosis. The issue of possible uneven distribution of patients with poorer prognosis could be better addressed by data modeling.

Considering other potential options for first-line treatment of patients with *ALK*-rearranged NSCLC, the combination of ALK-TKIs and immunotherapy was explored in several clinical trials with inconsistent results [134]. Preclinical studies showed that *ALK*-rearranged NSCLC cells may upregulate PD-L1 expression through activation of different signaling pathways [134]. Yet, the co-occurrence of PD-L1 expression in tumor cells and tumor-infiltrating lymphocytes, which could potentially be re-activated by ICIs, is rather infrequent in *ALK*-rearranged NSCLC [135]. This could be one explanation for the clinically observed low sensitivity of *ALK*-rearranged NSCLC to monotherapy ICIs [136]. Single-phase Ib trials combining ALK-TKIs and ICIs displayed some efficacy, albeit with significant limitations due to toxicity and variation in patients’ populations as well as study design [134].

To summarize, we are not able to define more precisely the patients in the three trials without clarifying the diagnostic intricacies and molecular profiles. Orthogonal tailored diagnostics, if available, should be prioritized for addressing reliability of detection and the heterogeneity of *ALK*-rearranged NSCLC [11,17,23,42,74,87]. It could enable the exploration of potential parallel therapeutic targets [137]. Furthermore, the nomenclature of *ALK*-rearranged NSCLC also deserves improved clarity. Tumors analyzed without NGS should principally be called ALK-IHC-positive and/or *ALK*-FISH-positive, while using the term “*ALK*-positive”—implicitly synonymous with “*ALK*-rearranged”—may be confusing if not confirmed by NGS.

To move forward, we need to revise the scientific questions in new clinical trials to address current challenges, such as defining different risk groups of *ALK*-rearranged NSCLC (Table 1 and Figure 2), which may influence the therapeutic decisions. Since advanced NSCLC is a devastating disease for most patients, there is still an urgent need to offer these patients an effective and tolerable medicine. Therefore, it is well rationalized that the ALTA-1L, ALEX, and CROWN studies predominantly focused on efficacy and safety. However, this might unintentionally have distracted the focus from the fact that *ALK*-rearranged NSCLC is a heterogenous disease and a median PFS patient with *ALK*-rearranged NSCLC is more hypothetical than real. In this respect, it would be clinically meaningful to better elucidate who in the population of *ALK*-rearranged NSCLC patients are the long-term responders and the early progressors.

## 6. Conclusions

- It is clinically important to better characterize a heterogenous disease such as *ALK*-rearranged NSCLC while choosing the first-line treatment.
- An optimized diagnostic approach should complementarily inform on the alterations in *ALK* and other genes.
- For this purpose, diagnostic biopsies of tissue should be examined comprehensively with histological assessment followed by ALK-IHC, *ALK*-FISH, and NGS (both DNA/RNA). Concordance among these methods should be considered. CtDNA at baseline should also be included.
- Up-front brain metastases, PS > 1, and/or presence of ctDNA represent unfavorable features linked to poorer outcomes.
- Merging the clinical, histological, and molecular features helps to define risk groups of *ALK*-rearranged NSCLC patients and direct treatment choice.

## Figures and Tables

**Table 1:** Favorable and unfavorable features for outcome (as objective response rate, progression-free survival, and/or overall survival) after first-line ALK-TKI treatment of patients with advanced *ALK*-rearranged NSCLC. IHC: immunohistochemistry; FISH: fluorescence in situ hybridization; NGS: next-generation sequencing; LUAD: lung adenocarcinoma; ctDNA: circulating tumor DNA. ctRNA: circulating tumor RNA. * squamous cell carcinoma, large-cell neuroendocrine carcinoma, small-cell lung carcinoma; PS: performance status.

	Favorable Features	Unfavorable Features	References
**1**	Concordant IHC, FISH, and NGS	Discordant IHC, FISH, and NGS	[9,10,11,12,13,14,15,16,17,18,19,20,21,22,23,24,25,26]
**2**	Pure LUAD	LUAD combined with other histological types *	[27,28,29,30,31,32,33,34,35,36,37,38,39]
**3**	Canonical *ALK* fusion (except *EML4-ALK* v.3)	Non-canonical *ALK* fusion, *EML4-ALK* v.3, double/triple fusions	[40,41,42,43,44,45,46,47,48,49,50,51,52,53,54,55,56,57,58,59]
**4**	High % of *ALK*-rearranged tumor cells by FISH	Low % of *ALK*-rearranged tumor cells by FISH	[13,14,15]
**5**	No de novo co-alterations	De novo co-alterations (*ALK*, *CDKN2A/B*, *EGFR*, *KRAS*, *MET*, *NF2*, *TP53*)	[40,41,57,58,59,60,61,62,63,64,65,66,67,68,69,70]
**6**	No ctDNA/ctRNA	ctDNA/ctRNA	[1,8,41,71,72,73,74,75,76,77,78,79]
**7**	No brain metastases	Brain metastases	[1,2,6,7,8,71,80,81,82,83,84,85,86,87,88]
**8**	PS 0–1	PS ≥ 2	[6,7,8,57,71,80,82,83,87,88,89]
**9**	Treatment with next-generation ALK-TKIs	Treatment with Crizotinib	[1,6,7,8,44,57,87,88,90]
